# Exploratory Investigation of Impact Loads During the Forward Handspring Vault

**DOI:** 10.1515/hukin-2015-0034

**Published:** 2015-07-10

**Authors:** Gabriella Penitente, William A. Sands

**Affiliations:** 1 Academy of Sport and Physical Activity, *Sheffield Hallam University, Sheffield, UK*; 2 Centre for Sport and Exercise Science, *Sheffield Hallam University, Sheffield, UK*

**Keywords:** upper extremity loading, gymnastics, kinetics, kinematics, injury

## Abstract

The purpose of this study was to examine kinematic and kinetic differences in low and high intensity hand support impact loads during a forward handspring vault. A high-speed video camera (500 Hz) and two portable force platforms (500 Hz) were installed on the surface of the vault table. Two-dimensional analyses were conducted on 24 forward handspring vaults performed by 12 senior level, junior Olympic program female gymnasts (16.9 ±1.4 yr; body height 1.60 ±0.1 m; body mass 56.7 ±7.8 kg). Load intensities at impact with the vault table were classified as low (peak force < 0.8 × body weight) and high (peak force > 0.8 × body weight). These vaults were compared via crucial kinetic and kinematic variables using independent t-tests and Pearson correlations. Statistically significant (p < 0.001) differences were observed in peak force (t(24) = 4.75, ES = 3.37) and time to peak force (t(24) = 2.07, ES = 1.56). Statistically significant relationships between the loading rate and time to peak force were observed for high intensity loads. Peak force, time to peak force, and a shoulder angle at impact were identified as primary variables potentially involved in the determination of large repetitive loading rates on the forward handspring vault.

## Introduction

Gymnastics is somewhat unique in that the athletes actually ‘jump’ from their hands as well as their feet. Clearly, jumping from one’s hands is more difficult and places extraordinary demands on limbs that were designed for reaching and grasping rather than jumping and landing. The inherent problem of using the upper extremities for jumping and landing has been recognized for some time in gymnastics (Beunen et al., 1999; Di Fiori et al., 2006).

In 2001, the International Gymnastics Federation changed the vaulting apparatus in order to facilitate performance and safety in men’s and women’s artistic gymnastics. The replacement of the vaulting horse with the vaulting table has been one of the most significant modifications to influence gymnastics tactics and performance. The necessity for a new apparatus was related to an increasing incidence of injury (Sands et al., 2003). The vaulting table maintained the traditional competition top surface height (1.25 m for women and 1.35 m for men), however, it is characterized by a completely different shape, geometry, and elasticity properties. The shape has been described as a ‘tongue’ shape, with a 40% wider and three times longer top surface than the previous women’s vaulting horse apparatus. Moreover, the upper surface of the table is slightly inclined (about 5°).

**Figure 1:**
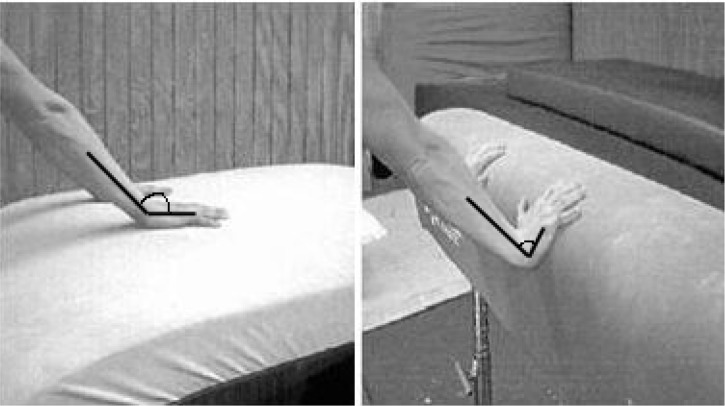
This picture is a demonstration of the hand placement. Vault table hand position for front handspring-type vaults on the horse vault (right) and table vault (left). Note that the wrist angle on the table vault surface appears less extended than on the horse vault (pictures modified with permission by Sands and McNeal, 2001).

The new vault table features listed above created numerous advantages for gymnasts. In particular, women gymnasts were able to benefit from a wider, longer and more visible surface thus reducing hand placement inaccuracy errors in the pre-flight phase (from a springboard to a vault table), improved confidence in the hand placement on the apparatus, and a softer and slightly elastic hand contact surface. The impact and push-off actions during the hand contact phase were thought to be enhanced by the changes provided by the vault table. Figure 1 shows typical forward handspring-style hand placement for an old vault horse and a current vault table. The table surface may enhance a wrist position by allowing a less severe hyper-extended position (Sands and McNeal, 2002).

A discourse on gymnastics nearly always turns to injury and injury prevention. Injury remains the most serious problem for gymnastics (Sands, 2000). Epidemiologic studies of gymnastics injuries have often found the vaulting event to be ranked the highest in terms of injury incidence and severity (Caine et al., 2003), and the wrist has been shown to be particularly vulnerable in both acute and over-use injuries (De Smet et al., 1994; Liebling et al., 1995; Sands et al., 1993). However, since the introduction of the vaulting table the incidence of upper extremity injuries does not appear to have decreased (Webb and Rettig, 2008), in fact, between 70 and 80% of the gymnasts still suffer from wrist injuries (Di Fiori et al., 2006). According to Singh et al. (2008), upper extremities account for 42% of the gymnastics injuries and handspring-type skills are most frequently associated with injuries. Although direct causation of wrist injuries associated only with vaulting is difficult to demonstrate due to the multi-event nature of women’s gymnastics, it is common to observe gymnasts performing their vaults with taped wrists or wearing protective wrist braces, and often train and compete with wrist pain (Beunen et al., 1999). An excessive loading pattern may also contribute to injuries at other locations such as an elbow, a shoulder and a neck (Sands et al., 1993; Wadley and Albright, 1993). For instance, indirect forces transmitted through outstretched and abducted arms (e.g., catching oneself from a forward fall to the hands) can drive the head of the humerus posteriorly and result in a posterior dislocation of the shoulder (Whiting and Zernicke, 1998). It has been suggested that upper extremity injuries such as sprains, strains, contusions, tendonitis, and bursitis are due to intense compressive loads generated at the hands during repetitive hand support impacts (Nattiv and Mandelbaum, 1993; Werner and Plancher, 1998).

A preliminary investigation on two-dimensional kinetic data collected from direct measurement during the contact phase of the gymnasts’ hands with the vault table showed possible injury-related factors (Penitente et al., 2010). Thus, the present study may find a rationale for urgency in understanding how the magnitude of hand support impact forces and accompanying kinematics may be linked to upper extremity trauma. Results from this study may also provide preliminary information that will assist physiotherapists and orthopaedists in return-to-activity decisions.

The main purpose of the present exploratory study was to test the hypothesis that the impact events with the table that were characterized as high intensity (HI, forces with impact peaks > 0.8 body weight (BW)) were associated with potential upper extremity injury risk factors. We also hypothesized that associated risk factors were: shorter time to impact peak force, a larger loading rate, a greater impulse load, greater wrist hyperextension, greater shoulder extension angles, and a greater centre of mass vertical velocity at hand contact. In addition, we hypothesized that the variables above would contrast statistically with forward handsprings executed with low intensity (LI, forces with impact peaks < 0.8 BW).

## Material and Methods

### Participants

Twelve level 10 junior Olympic national team female gymnasts with a mean age of 16.9 ±1.4 yr, body height of 1.60 ±0.1 m and body mass of 56.7 ±7.8 kg volunteered for this study. USA gymnastics classifies these gymnasts immediately below the international competitive levels. Gymnasts provided informed consent and ethical approval was granted in accordance with the United States Olympic Committee policies on research at the United States Olympic Training Center.

### Measures

A video camera (500 Hz, Photron 1280, Motion Engineering Company, USA) was positioned on the side of the table with its optical axis perpendicular to the direction of the movement. The recorded videos were scaled by means of a rectangular calibration frame measuring 1.00 × 1.10 m, used for two-dimensional (2D) kinematic analyses of eleven reflective markers (diameter 22.5 mm) (5^th^ metatarsal joint, calcaneus, lateral malleoulus, lateral condyle, greater trochanter, inferior lateral angle of the 12^th^ rib, shoulder, lateral epicondyle, ulnar styloid, 5^th^ metacarpal joint, and head). The markers were used to identify a nine-segment body model. Markers were digitized using Peak Motus^TM^ 9.1 (Peak Performance Technologies, USA). The position of the calibration frame encompassed the space used by the gymnasts during the hand-table contact phase. Coordinates were smoothed using a Butterworth digital filter with frequency cut-off between 5 and 8 Hz.

The centre of mass (CM) was calculated using the Kjeldsen’s model of female gymnasts (Plagenhoef, 1971). The orientation of the 2D system had the x-axis aligned along the main horizontal direction of movement and the z-axis aligned vertically. The following kinematic variables were selected: a wrist angle, a shoulder angle and CM horizontal and vertical velocities at hand-table impact. The wrist joint angle was identified as the relative angle in the sagittal plane of the forearm and the hand segments (the wrist angle of 180° corresponded to a position with the forearm and hand aligned; Figure 1); the shoulder angle was identified as the anterior relative angle in the sagittal plane of the trunk and the upper arm segments (the shoulder angle of 180° corresponded to a position with the trunk and upper-arm aligned).

### Procedures

**Figure 2:**
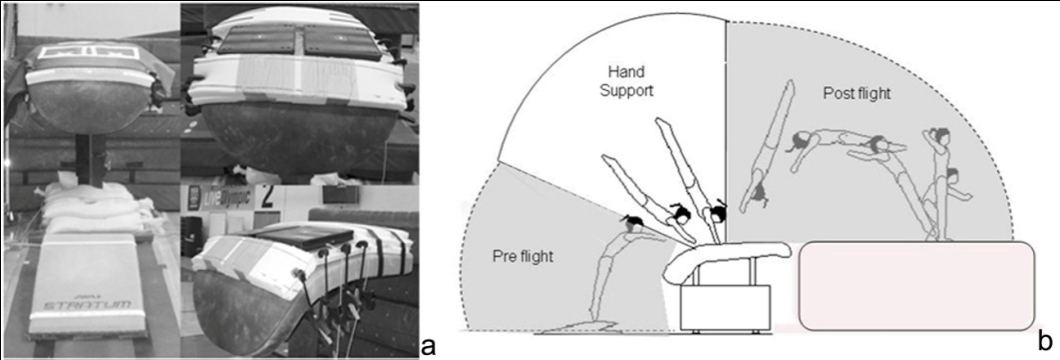
2a-Two portable force platforms mounted on a plywood based, secured to the table and covered with a thin mat. The taped lines on the mat surface designed the edges of the force platforms to provide a visual target for the gymnasts’ hands placement; (left). 2b - Forward handspring vault drill (right): Pre-flight (from springboard take-off to hand-table impact); Hand Support (from hand-table impact to hand-table take-off); Post-flight (from hand-table take-off to feet-mat impact). Only the Hand support phase (white section in the picture) was analyzed in the present study.

The vault table surface was equipped with two portable force platforms 37 × 37 × 4.5 cm (Pasco Scientific, USA) fixed to a rigid wooden foundation base. The force platforms were covered with a thin mat to ensure cushion and traction during hand contact (0.4 cm) and the edges of the force platforms were designated by taped lines placed on top of the thin mat surface to provide visual targets for the gymnasts’ hand placements (Figure 2a). The vault table was set at the women’s competition height of 1.25m. Reaction forces generated during forward handspring vaults were measured in the vertical (Z) and anterior-posterior (X) planes at a rate of 500 Hz. The accuracy of each force platform mounted on a rigid wooden foundation was calibrated via static linearity (both vertical and horizontal components), static regionality, and dynamic force-time comparisons against a laboratory force platform with known validity (Penitente et al., 2010).

Gymnasts participated in a self-selected warm up activity before performing a forward handspring vault landing feet-first on mats stacked to the level of the vault table (Figure 2b). Twenty-four successful trials were selected (two for each gymnast) including a simultaneous measurement of left and right hands from the two force platforms. In order to combine kinematic and kinetic variables only the 24 impact events recorded from the right hand were used for analysis.

### Statistical Analysis

Forces were scaled to each gymnast’s body mass. The following kinetic variables were investigated: impact (Fz) and braking (Fx) peak force magnitudes (BW), time from contact to vertical (Fz) and braking (Fx) peak force (s), a loading rate (from contact to impact peak force -Fz) (BW·s^−1^) [24], a vertical impulse (BW·s), and a horizontal impulse (BW·s).

**Figure 3:**
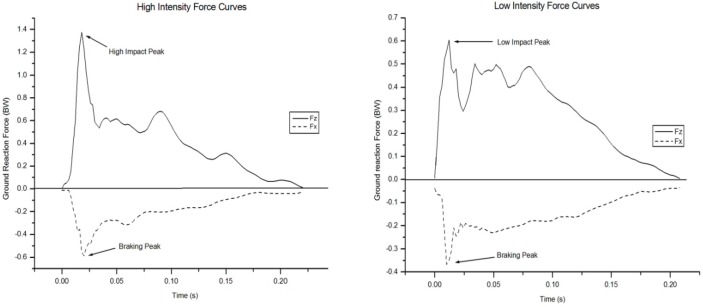
Sample, hand-support phase force-time data for the High (left) and Low (right) Load Intensity groups. The continuous and dashed lines represent the vertical (Fz) and anterior-posterior (Fx) forces, respectively.

Based on the split median method, data were divided in two groups. The first group was formed by those forward handsprings that showed impact peak force magnitudes less than 0.8 BW (LI group), operationally defined as ‘low intensity load’. The second group was determined by impact peak force greater than 0.8 BW (HI group), operationally defined as ‘high intensity load’ (Markolf et al., 1990) (Figure 3).

Data analyses were performed with the software SPSS 18.0 (SPSS, Inc. Chicago, USA). The reliabilities between the two trials performed by each gymnast were assessed by intra-class correlation coefficients (ICCs) (alphas ranged from 0.26 to 0.85). Some variables indicated marked individual variances that were not always captured by the ICCs and some variables showed as high as 20% relative error between performance trials. Due to the exploratory nature of this study and in the attempt to maintain a degree of acknowledgement of a marked individual variability of the athlete performance, the trials variables were not collapsed to produce a single mean for each athlete. Moreover, the fact that such variability occurred is considered an important aspect of this study’s data (Bates, 1996). All the variables were tested for normality according to the Shapiro-Wilks procedure. Differences in kinetic and kinematic variables between HI and LI were assessed with the independent t-test using both trials for each gymnast (p < 0.05). As both trials for each gymnast were used for analysis, the comparisons between HI and LI were tested using the method described by Gönen et al. (2001) that accounts for within subject clustering. Thus, the t statistic was divided by a correction factor defined as C = [1 + (m − 1)ϱ], where m is the number of trials for a gymnast and ϱ is the intracluster correlation (ϱ = Variance between subjects / Variance between subjects + Variance within subjects). The Cohen’s d effect size index was used to estimate the magnitude of significant differences between HI and LI groups (Cohen, 1988). Pearson’s correlation (p < 0.05) was used to determine the relationships among the kinetic and kinematic variables.

## Results

The force peak magnitude of the twenty-four trials indicated that twelve trials were LI impact load and twelve were HI impact load. The descriptive statistics relative to the kinetic and kinematic variables for LI and HI groups are presented in Table 1.

Impact peak force (t(24) = 4.75, p < 0.001) and time to impact peak (t(24) = 2.07 p < 0.001) were the only variables showing a statistically significant difference between HI and LI groups. Further, Cohen’s d values (3.37 and 1.56, respectively) indicated a large effect size.

The HI group showed a statistically significant correlation between the time to impact peak and the loading rate (r = −0.78, p = 0.003), the time to braking peak (Fx) (r = 0.83, p = 0.001), the CM horizontal velocity at hand impact (r = 0.82, p = 0.047), and CM horizontal velocity with the wrist angle at hand impact (r = −0.63, p = 0.027). The loading rate resulted in a statistically significant relationship with the time to braking peak force (r = −0.82, p = 0.001) and the wrist angle at impact (r = 0.73, p = 0.007). The braking peak force showed a statistically significant relationship with the horizontal impulse (r = −0.64, p = 0.024). The shoulder angle at hand impact was significantly correlated with the wrist angle at the same instant of impact (r = 0.62, p = 0.032).

The LI group showed a statistically significant correlation between the impact peak force and the loading rate (r = 0.67, p = 0.017). The time to impact peak force and the CM horizontal velocity at impact were statistically correlated (r = 0.74, p = 0.006). The time to braking peak force was statistically correlated with the horizontal impulse (r = −0.75, p = 0.005). The shoulder angle at hand impact showed a significant correlation with the time to braking peak force (r = −0.73, p = 0.007) and with the horizontal impulse (r = 0.67, p = 0.018).

## Discussion

This study was designed to investigate the intensity of impact loads obtained during the forward handspring vault performed by highly trained female gymnasts. Second, the study was aimed to determine the magnitudes and interactions among kinetic and kinematic variables that characterize hand-table impact events and duration with high and low intensity loads.

The magnitude of compressive impact, the loading rate (Nigg, 1985), the impulse, the angular position of the wrist and shoulder at hand support impact, and the centre of mass velocities have been identified as primary contributors to upper extremity trauma (Caine et al., 2003; De Smet et al., 1994; Liebling et al., 1995; Sands et al., 1993). The forward handspring skill was chosen as standard fundamental skill commonly used by coaches to develop higher scoring performances and, for research in safety issues.

Major findings indicated that the two intensity groups identified were characterized by statistically significant differences in impact peak force magnitude and time to impact peak force; however, no statistically significant differences in the overall loading rate were observed. The rate at which upper and lower extremities are loaded has been implicated in stress fractures and soft tissue dysfunctions (Nigg, 1985; Markolf et al., 1990; Seeley and Bressel, 2005). From an injury risk perspective, the results from the present study indicate that during the handspring vaults, the shock absorption demands placed on the upper extremities are high, particularly when extrapolated to dozens of daily repetitions.

This is the first study to directly measure the reaction forces during the hand support of a gymnastics vault. As there are no measurements of the impact loading rate associated with similar skills in the literature, a direct comparison of our results with other studies cannot be made. However, if we consider forward handspring skills as a particular ‘form of a take-off’ or a ‘jump’ that involves hands rather than feet, comparisons with lower extremity jump exercises can be made. Results by Richard and Veatch (1994) showed that loading rates of the lower extremities could be categorized as high during hopping-type jumps from different jumping heights. It is interesting to note that the loading rates observed for the forward handsprings with LI loads (68.2 BW.s-1) were greater than the loading rates produced during lower extremity drop jumps from a height of 6 cm (56.99 BW.s-1). The loading rate found for the HI load group (96.1 BW.s-1) was greater than the loading rate developed during a drop jump from a height of 8 cm (73.1 BW.s-1) (Richard and Veatch, 1994). The maximum loading rates recorded for both groups (LI = 151.4 BW·s-1 and HI = 161.6 BW·s-1) were greater than that associated with each leg during a two-foot landing drop jump from a height of 61 cm (136 BW·s-1) measured by Bauer et al. (2001). Moreover, in the HI load group in the present investigation, the impact peak force was characterized by magnitudes comparable with typical impact force generated during running at 3 m·s-1 (1.6 ± 0.4 BW) (Munro et al., 1987).

In upper extremity stretching-shortening-type motions such as the forward handspring, there are large and relatively unnatural ranges of impact loads similar in magnitude to the lower extremities; the risk of injury is obviously high (Markolf et al., 1990). The vertical forces observed during the present study in HI handspring vaults may be intense enough alone or in aggregate to cause injuries (such as distal radial syndrome, carpal stress fracture, capsulitis, positive ulnar variance and carpal instability) associated with weight-bearing gymnastics exercises in general (Gabel, 1998). Werner and Plancher (1998) reported that 90% of wrist injuries are related to compressive stress, and closely related to this type of stress is a loading rate (Markolf et al., 1990).

A comparison between the impact peak forces and loading rates measured in the present study with those measured by Roy et al. (1985) during two gymnastics tumbling skills, round-off on the floor (impact peak = 2.2 ± 0.3 BW; loading rate = 19.2 ± 4.6 BWs-1) and round off on the vaulting springboard (impact peak = 2.4 ± 0.3 BW; the loading rate = 28.6 ± 6.7 BW·s-1). In the tumbling skills analysed by Roy et al. (1985), the higher impact loads in the round-off are associated with lower loading rates. In contrast, the present study shows that both intensity groups displayed high loading rate values during hand contact with similar CM velocities. These results contrast with the assumption that impact peak force and a loading rate are speed-dependent, as shown in running activities (Munro et al., 1987), it is not applicable to handspring vault hand support skills. In addition, the premise that high impact forces accompany high loading rates in jumping movements (McNitt-Gray, 1991) is not similarly associated with vault handspring skills. In fact, this study showed that low impact peak forces may produce high loading rates. This was supported by the absence of a significant correlation between hand-table impact peak forces and loading rates.

For the HI group, the loading rate was related to the time to vertical peak force. A short time to peak force (0.007 ± 0.003 s LI; 0.016 ± 0.008 s HI) appeared to be more likely a crucial factor in generating high loading rates and thereby may be related to injury potential. A similar finding was reported by Dixon and Kerwin (1999) in their study on the influence of a heel lift on the Achilles tendon load during running. It is important to consider that the time to impact peak is related to muscle pre-activation which is used to control and attenuate or accentuate impact loading (Nigg, 1985). It has been shown that subjects’ ability to prepare their bodies for shock absorption depends on factors such as time, segment kinematics, tissue compressibility and elasticity, and vision preceding the impact. It was suggested that these components can affect muscle activation prior to contact, and in turn influence vertical peak force magnitude and impulse duration (Nigg, 1985). Muscle pre-activation characteristics may explain the differences in impact peak forces and times to impact peak between HI and LI groups. McNeal and colleagues (2007) showed that muscle activation timing and magnitude were related to take-off kinetics and kinematics in tumbling take-offs. In contrast with our hypothesis, the time to reach the impact peak was longer for the HI group. This may be due to the weaker push action of the LI group. The weaker push was observed from a qualitative analysis of the performance trials. It was noted that gymnasts of the LI group appeared to ‘pull’ or ‘release’ their hands from the table rather than push against it.

The LI group showed positive correlations between shoulder angles at hand contact and a braking impulse. Regarding technique, a statistical positive relationship between a shoulder angle and a breaking and vertical impulse in the forward handspring on the floor has been identified as a performance factor influencing the ‘blocking effect’ (i.e. rapid push from the hands) at impact. Impact events with poor shoulder flexion have been associated with dissipation of ground reaction force (Nelosn and Metzing, 1995).

Finally, the wrist and shoulder angles did not show significant differences between HI and LI groups. However, for HI impacts the relationships of the wrist with the shoulder angles, the times to impact peak forces and the loading rates demonstrated that gymnasts who approached the apparatus with the wrist more hyper-extended also had the shoulder more flexed, reached the impact peak slower and developed a lower loading rate. These results confirm that while the wrist angle at hand contact did not show any obvious direct relationship with hyperextension injury in relation to compressive load, the shoulder angle may be seen as a critical injury factor (Sands et al., 1993; Wadley and Albright, 1993; Whitinh and Zernicke, 1998). It could be suggested that the shoulder angle at impact may play a role in determination of time to impact peak and thus of the magnitude of the loading rate.

Limitations in this study were primarily due to the exploratory-descriptive nature of the investigation. However, this is the first study to identify and characterize crucial kinetic and kinematic variables as potential injury contributors through direct measurement of the hand-table impact events on the gymnastics vaulting table. The findings obtained represent a valuable starting point to develop other investigations involving male gymnasts and more complex vault types.

## Conclusions

High loading rates were found for both high and low intensity impact events. Results show that the short time to impact peak in conjunction with the position of the shoulder may be a likely contributor to injurious loading rates in addition to high impact peak forces.

Significant relationships between the loading rate and time to peak force were observed for high intensity loads. Peak force, time to peak force, and a shoulder angle at impact were identified as primary variables potentially involved in the determination of large repetitive loading rates on the forward handspring vault.

## Practical Implications

Based on the findings of the present study it can be recommended to coaches that they encourage a rapid repulsive action and a shoulder position at full flexion in line with the torso. This study also suggests combining the practice of vaulting skills in combination with a specific flexibility and conditioning program in order to build stronger and more reactive upper extremity skill and strength. Finally, to completely understand the injury mechanisms during the vault exercise it will be necessary to investigate other intrinsic and extrinsic performance factors. For instance, further investigations of the elastic characteristics of the table surface are necessary to show if the vault table enhances the gymnast’s ability to basically take-off (i.e. jump) from the hands.

## Figures and Tables

**Table 1:** Forward Handspring vault kinetic and kinematic characteristics

		N	Mean (SD)	Range
Impact Peak - Fz (BW)	Low Load	12	0.46 (0.18)^*^	[0.15 – 0.74]
	High Load	12	1.37 (0.34)^*^	[0.86 – 1.81]
				
Time to Impact Peak - Fz (s)	Low Load	12	0.007 (0.003)^*^	[0.004 – 0.012]
	High Load	12	0.016 (0.008)^*^	[0.008 – 0.030]
			
Loading Rate - Fz (BW·s^−1^)	Low Load	12	68.24(36.01)	[23.49 – 151.40]
	High Load	12	96.12 (38.75)	[49.94 – 161.60]
				
Vertical Impulse - Fz (BW·s)	Low Load	12	0.10 (0.009)	[0.088 – 0.120]
	High Load	12	0.11 (0.016)	[0.086 – 0.136]
				
Braking Peak - Fx (BW)	Low Load	12	−0.65 (0.14)	[−0.90 – −0.44]
	High Load	12	−0.61 (0.15)	[−0.95 – −0.342]

Time to Braking Peak - Fx (s)	Low Load	12	0.021 (0.008)	[0.006 – 0.034]
	High Load	12	0.015 (0.007)	[0.004 – 0.026]

Horizontal Impulse - Fx (BW·s)	Low Load	12	0.004 (0.008)	[−0.012 – 0.016]
	High Load	12	0.004 (0.005)	[−0.002 – 0.012]

Wrist angle at Impact (°)	Low Load	12	157.85 (9.29)	[144.04 – 174.41]
	High Load	12	156.57 (7.53)	[146.26 – 171.77]

Shoulder angle at Impact (°)	Low Load	12	131.62 (12.63)	[114.22 – 149.63]
	High Load	12	139.66 (7.87)	[126.62 – 148.26]

CM Hor Vel at Impact (m·s^−1^)	Low Load	12	2.28 (0.31)	[1.86 – 2.77]
	High Load	12	2.32 (0.29)	[1.81 – 2.82]

CM Vert Vel at Impact (m·s^−1^)	Low Load	12	4.09 (0.44)	[3.25 – 4.65]
	High Load	12	4.08 (0.40)	[3.49 – 4.93]

* Independent t-test test sign (p<0.05)

N indicates the number of trials characterized by Low and Hi Intensity Load.

‘Impact’ defined as the first frame of hand-table contact.
